# Influencing factor analysis and prediction model construction of dupilumab treatment adherence: a prospective cohort study in moderate-to-severe atopic dermatitis

**DOI:** 10.3389/fimmu.2025.1682777

**Published:** 2026-01-05

**Authors:** Pingxiang Ouyang, Siyu Yan, Jinrong Zeng, Lihua Gao, Lina Tan, Jianyun Lu

**Affiliations:** Department of Dermatology, The Third Xiangya Hospital, Central South University, Changsha, China

**Keywords:** atopic dermatitis, dupilumab, biologics, treatment adherence, Cox proportional hazards regression, machine learning

## Abstract

**Background:**

The efficacy of dupilumab in atopic dermatitis (AD) has been widely validated; however, systematic investigations into treatment adherence are lacking.

**Objective:**

To analyze clinical factors influencing dupilumab adherence in patients with moderate-to-severe AD and develop a multidimensional adherence prediction model to support precision management of biologic therapies.

**Methods:**

Using a single-center prospective cohort, a three-stage modeling approach was applied: (1) univariable Cox proportional hazards regression to identify potential predictors; (2) XGBoost modeling with SHAP method for feature importance ranking and dimensionality reduction; (3) multivariable Cox proportional hazards model for final prediction.

**Results:**

Univariable analysis indicated that treatment discontinuation was significantly associated with age, sex, combination therapy, baseline disease activity, and treatment response. Machine learning identified EASI/NRS and EASI-75/SLS-75 as key predictors of baseline disease activity and treatment response, respectively. The multivariable model confirmed independent predictive value for age, baseline EASI/NRS scores, and achievement of EASI-75/SLS-75.

**Conclusion:**

This study identified key determinants of dupilumab adherence and developed a predictive adherence model that offers personalized risk visualization via nomograms, providing an evidence-based tool for the precision management of AD biologic therapies.

## 1 Introduction

Atopic dermatitis (AD), a chronic inflammatory skin disease characterized by intense pruritus and barrier dysfunction, substantially compromises patients’ quality of life (1, 2). AD causes substantial public health burdens through medical costs and productivity losses, ranking among the most burdensome dermatological conditions (3). While typically emerging in infancy, AD frequently persists into or starting from adulthood, affecting approximately 10% of children and 7% of adults in US (4, 5). Its pathogenesis involves dysregulated Th2 immunity with IL-4/IL-13 activation and epidermal barrier defects (6–8). These immune and barrier abnormalities result in inadequate long-term control with conventional therapies in moderate-to-severe disease (9).

Dupilumab, a biologic blocking IL-4/IL-13 via IL-4Rα, has transformed AD management (10, 11). Phase III trials confirm its superior efficacy over placebo in moderate-to-severe AD, with comparable safety and fewer severe adverse events (12, 13). Guidelines recommend a 16-week induction phase followed by personalized maintenance (14–16). Despite efficacy, patient adherence remains challenging. Real-world evidence shows premature discontinuation after symptom control may cause recurrence (17, 18). Existing studies primarily treat adherence as a secondary endpoint, comparing profiles rather than exploring influencing factors (19, 20). Studies on treatment adherence in AD indicate that beyond cost and side effects, factors like forgetfulness, complex regimens, time burden, and poor disease understanding may also reduce adherence, leading to either unintentional or intentional therapy discontinuation (21, 22). Additionally, patient-specific circumstances, including socioeconomic status, occupational constraints, language preferences, cultural practices, and personal beliefs, may also impact adherence (23). These factors frequently lie beyond the scope of routine clinical assessment, making them difficult to comprehensively incorporate into clinical decision-making. Consequently, from a clinical perspective, developing methods to predict dupilumab adherence would valuably guide treatment optimization.

This prospective cohort study analyzes associations between clinical characteristics, including baseline disease activity and treatment response, and dupilumab adherence in moderate-to-severe AD. It aims to identify predictors of treatment discontinuation, construct a risk prediction model, and create individualized management strategies to optimize biologic therapy.

## 2 Materials and methods

### 2.1 Study population and ethical approval

Data came from an IRB-approved cohort study (#R23004) following Helsinki Declaration principles. The study analyzed a dynamic follow-up cohort of moderate-to-severe AD patients who initiated dupilumab treatment in the dermatology department and consented to data collection. The cohort recorded baseline characteristics and treatment responses at each follow-up visit. Due to varying follow-up durations and intervals, we used baseline and latest follow-up data from patients enrolled between June 2022 and June 2025 for retrospectively analysis to enable effective comparisons. Inclusion criteria were: (1) SCORAD≥25; (2) age≥6 years; (3) IGA≥3 after systemic therapies; (4) complete baseline and ≥2 follow-up records. Exclusion criteria were: (1) primary non-response; (2) development of severe adverse reactions; (3) development of severe complications or comorbidities. Based on these inclusion and exclusion criteria, 307 cases were ultimately included.

### 2.2 Outcome measures

The primary outcome was treatment discontinuation without medical indication, defined as patient-initiated cessation of dupilumab against physician recommendation, with treatment duration as secondary outcome. Comorbidities were defined as the presence of ≥1 diagnosed condition among allergic rhinitis, asthma, urticaria, or conjunctivitis. Family history required AD or these comorbidities in first-/second-degree relatives. Combination therapy included concurrent use of other AD medications under medical supervision, such as antihistamines or glucocorticoids. While all patients started with biweekly dosing after initially doubling dose, some transitioned to extended dosing intervals during long-term treatment in consultation with their physicians. Therefore, the latest follow-up dosing interval was used for analysis, reflecting real-world practice and enhancing model applicability. Comorbidities, combination therapy and dosing interval details are in **Supplementary Table 1**.

Using age-dependent assessment tools, such as CDLQI for patients under 16 years, 12 participants switched assessment scales. Efficacy was evaluated under double-blind protocol by two dermatologists with at least five years of experience in AD management independently assessing investigator-assessed metrics, including Investigator’s Global Assessment (IGA), Body Surface Area (BSA), Eczema Area and Severity Index (EASI), SCORing Atopic Dermatitis (SCORAD) (24–27). Mean scores were calculated from independent evaluations. Patient-reported outcomes (PROs) were collected through self-administered standardized scales, comprising Dermatology Life Quality Index/Children’s Dermatology Life Quality Index (DLQI/CDLQI), Patient-Oriented Eczema Measure (POEM), Numeric Rating Scale (NRS) for pruritus, Sleep Loss Score (SLS) in SCORAD, Atopic Dermatitis Control Tool (ADCT) (28–32), with researchers providing semantic clarification. Treatment response assessment system used 75% improvement thresholds, such as EASI-75, BSA-75, and SCORAD-75, to analyze response-duration relationships (33).

### 2.3 Study design

Study variables comprised three categories: basic medical characteristics, baseline disease activity scores, and final treatment response. Cox proportional hazards regression, a statistical method widely applied in survival analysis, was employed as the primary analytical framework (34). Due to the known correlations among disease activity scores, which would introduce multicollinearity in multivariable Cox proportional hazards model (variance inflation factor, VIF>5), a three-stage modeling strategy was implemented to identify robust predictors of dupilumab treatment discontinuation and guide clinical decision-making. First, all potential predictors underwent screening through univariable Cox proportional hazards regression. Second, significant clinical predictors with multicollinearity were stratified into four dimensions: baseline investigator-assessed metrics, baseline PROs, investigator-evaluated improvement endpoints, and patient-reported improvement endpoints. Machine learning then ranked feature importance within each dimension. Finally, the top predictor from each dimension was selected for multivariable Cox proportional hazards regression, along with significant basic medical characteristics. The study design is summarized in the Graphical Abstract.

### 2.4 Statistical analysis

Continuous variables are summarized as median [Q1-Q3], categorical variables as frequency [n (%)]. Non-normally distributed continuous variables with unequal variances (per Shapiro-Wilk and Levene’s tests) were compared between-group using Wilcoxon rank-sum test. Categorical variables were analyzed with Fisher’s exact/Chi-square tests. Statistical significance was set at *P*<0.05 (two-tailed).

Cox proportional hazards regression used treatment discontinuation without medical indication and treatment duration as dependent variables to calculate the hazard ratio (HR) and its 95% confidence interval (CI) for each independent variable. Statistical significance was set at *P*<0.05 (two-tailed). Nomograms visualized the model. Harrell’s concordance index (C-index) evaluated model discriminative capacity, while 1000 bootstrap calibrations assessed prediction-observation agreement (35). Model goodness-of-fit and potential overfitting were evaluated with Cox-Snell R² and Nagelkerke R² (36). Minimum sample size for developing the multivariable prediction model was calculated according to Riley et al.’s methodology (37, 38).

A structured procedure was implemented for the development and optimization of the best machine learning model. First, the dataset was randomly divided into training and testing subsets at a 7:3 ratio, using decision tree as the base learner. XGBoost was applied for model construction (39), with hyperparameter optimization conducted through comprehensive grid search. Then, the model performance was evaluated using the area under the receiver operating characteristic (ROC) curve (AUC) derived from 10-fold cross-validation (40). Finally, SHapley Additive exPlanations (SHAP) allocated the overall prediction changes to each feature, enabling quantification of each feature’s relative importance and the directional impact of its values on model predictions (41, 42). Pearson correlation assessed feature-SHAP value relationships across the entire sample (43).

All statistical analyses were performed in R (v4.4.2) using the “pmsampsize” package (v1.1.3), “survival” package (v3.6-4), “xgboost” package (v1.7.8.1), “caret” package (v7.0-1), and “shapviz” package (v0.9.7).

## 3 Result

### 3.1 Overview of the study population

This study analyzed 307 moderate-to-severe AD patients treated with dupilumab, with a median treatment duration of 413.0 days (Q1-Q3: 364.5-468.0). Ninety-four patients unilaterally discontinued treatment against physician recommendation, forming the treatment discontinuation group, with most becoming lost to follow-up. While the remainder constituted the continued treatment group, which included both active treatment recipients and those who discontinued under medical guidance. Initial comparison of overview characteristics between groups revealed notable differences (**Table 1**). However, establishing causal relationships requires incorporation of temporal variables within the analytical framework, as this approach helps filter out false positive results attributable to temporal heterogeneity. For example, while significant between-group differences in treatment intervals were observed (*P*<0.001), subsequent Cox regression indicated that treatment interval was not a significant predictor of adherence (*P* = 0.373). Baseline disease activity scores confirmed that all enrolled patients met diagnostic criteria for moderate-to-severe AD (IGA ≥3, or BSA ≥10%, or SCORAD ≥25) (44).

**Table 1:** Overview characteristics of the study population.

Characteristics	Treatment discontinuation (N = 94)	Continued treatment (N = 213)	P-value
Age (years), median [Q1-Q3]	15.0 [10.0-22.8]	34.0 [20.0-60.0]	< 0.001
Age group, n (%)			
6 to < 18 y	64 [68.1]	36 [16.9]	< 0.001
18 to < 60 y	21 [22.3]	123 [57.7]	
≥ 60 y	9 [9.6]	54 [25.4]	
Age at AD onset (years), median [Q1-Q3]	3.5 [1.0-11.0]	24.0 [10.0-45.0]	< 0.001
Male, n (%)	75 [79.8]	113 [53.1]	< 0.001
Family history, n (%)	57 [60.6]	111 [52.1]	0.167
Comorbidity, n (%)	53 [56.4]	96 [45.1]	0.068
Combination therapy, n (%)	26 [27.7]	93 [43.7]	0.008
Treatment interval (weeks), median [Q1-Q3]	3.0 [2.0-4.0]	2.0 [2.0-3.0]	< 0.001
Severity scores of AD at the initial visit, median [Q1-Q3]			
IGA [0-4^#^]	3.0 [3.0-4.0]	3.0 [3.0-4.0]	0.365
BSA (%) [0-100^#^]	28.0 [19.0-48.8]	36.0 [20.0-56.0]	0.279
EASI [0-72^#^]	10.0 [7.5-16.6]	13.5 [7.6-27.7]	0.093
SCORAD [0-103^#^]	41.6 [32.7-54.2]	51.2 [41.5-66.5]	< 0.001
DLQI^*^/CDLQI^**^ [0-30^#^]	9.0 [6.0-13.0]	15.0 [11.0-20.0]	< 0.001
POEM [0-28^#^]	13.0 [10.0-18.0]	18.0 [14.0-22.0]	< 0.001
NRS [0-10^#^]	6.0 [4.0-7.0]	8.0 [7.0-10.0]	< 0.001
SLS [0-10^#^]	4.0 [3.0-6.0]	8.0 [5.0-9.0]	< 0.001
ADCT [0-24^#^]	10.0 [6.3-17.0]	18.0 [13.0-21.0]	< 0.001
Improvement of severity scores at the last visit, n (%)			
IGA 0/1^+^	8 [8.5]	25 [11.7]	0.40
BSA-75^++^	63 [67.0]	108 [50.7]	0.008
EASI-75^++^	78 [83.0]	113 [53.1]	< 0.001
SCORAD-75^++^	4 [4.3]	46 [21.6]	< 0.001
DLQI-75^++^/CDLQI-75^++^	21 [22.3]	63 [29.6]	0.190
POEM-75^++^	14 [14.9]	75 [35.2]	< 0.001
NRS-75^++^	5 [5.3]	72 [33.8]	< 0.001
SLS-75^++^	10 [10.6]	102 [47.9]	< 0.001
ADCT-75^++^	8 [8.5]	85 [39.9]	< 0.001
Treatment duration (days), median [Q1-Q3]	358 [326.8-385.5]	434.0 [403.0-484.0]	< 0.001

### 3.2 Univariable Cox proportional hazards model

**Figure 1:**
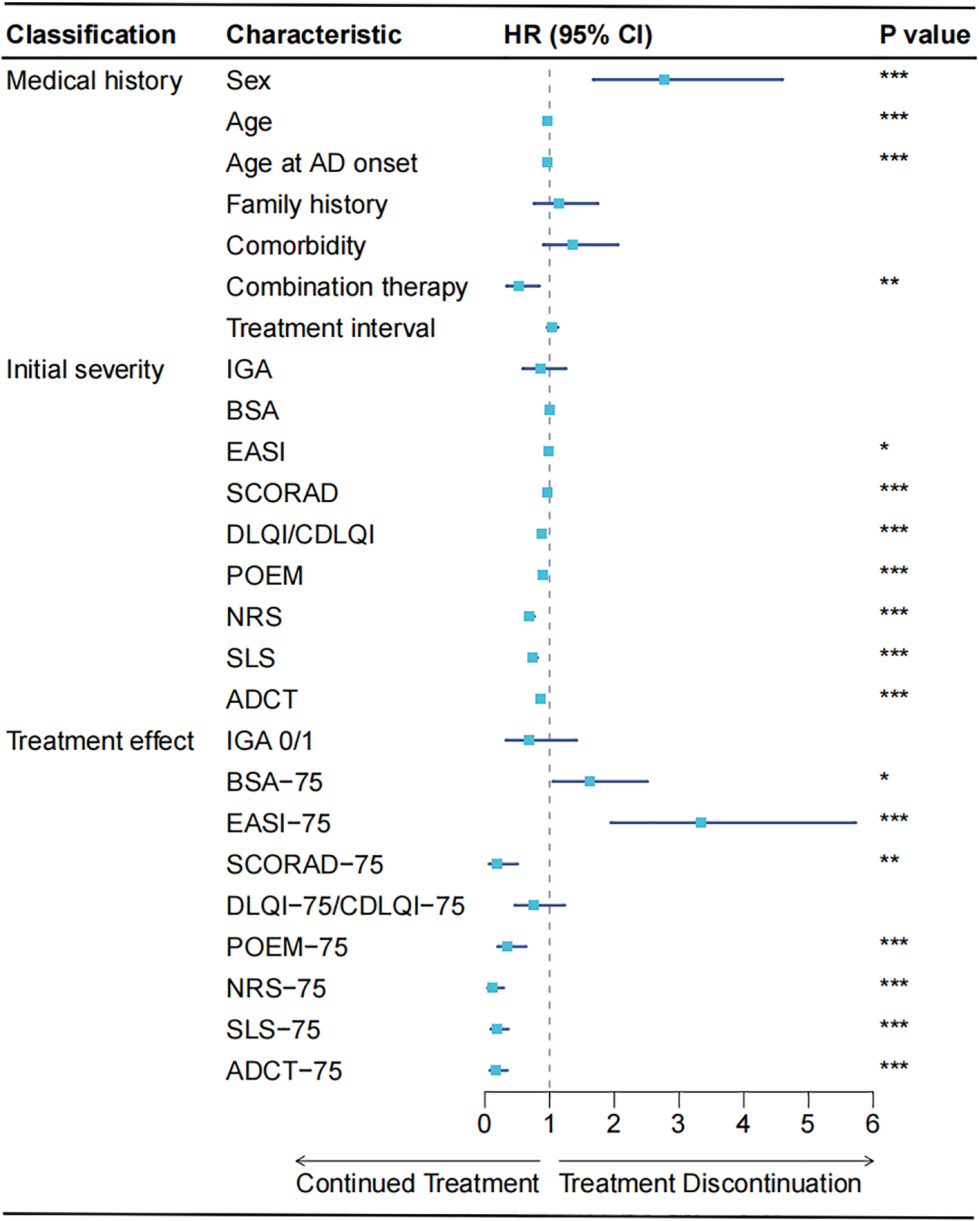
Atopic dermatitis. Univariable Cox proportional hazards model for the correlation analysis between single variables and treatment discontinuation. The forest plot displays the hazard ratios (HRs) with 95% confidence intervals (95% CIs). For Sex, males were assigned a value of 1 and females 0; for Comorbidity and Combination therapy, presence was coded as 1 and absence as 0. *P <0.05, **P <0.01, ***P <0.001.

Univariable Cox regression identified male sex, younger age, and absence of combination therapy as significant predictors of treatment discontinuation (**Figure 1**, **Supplementary Table 2**). Comorbidities, family history, and treatment interval showed no significant associations with treatment discontinuation. Lower baseline disease activity scores predicted higher discontinuation risk. Failure to achieve PROs improvement increased discontinuation risk, while achieving investigator-assessed EASI-75/BSA-75 paradoxically increased risk. SCORAD-75 achievement showed a protective effect, likely explained by its composite design incorporating both investigator assessments and patient-reported symptoms.

A particularly notable paradoxical finding emerged from the analysis: achievement of EASI-75 was significantly associated with reduced treatment adherence in both Wilcoxon rank-sum test (**Table 1**) and Cox regression analysis (**Figure 1**). This pattern contrasts sharply with improvement endpoints incorporating PROs, including SCORAD-75, POEM-75, NRS-75, SLS-75, and ADCT-75, all of which showed significant positive correlations with improved adherence.

### 3.3 Machine learning model

**Figure 2:**
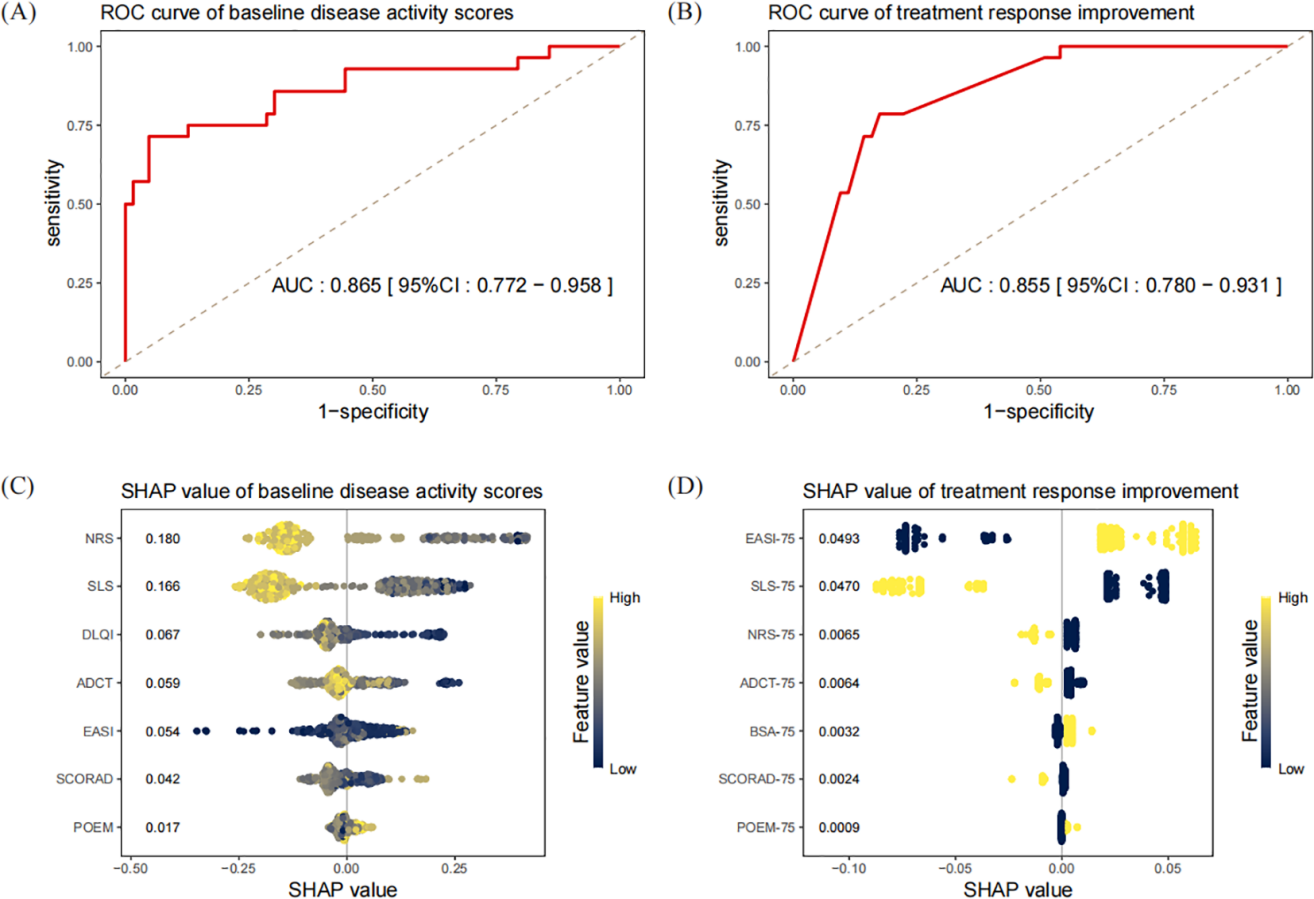
Atopic dermatitis. ROC curves and SHAP summary plots of XGBoost for predicting of treatment discontinuation. Each point in the summary plot represents the SHAP value of one sample, with the y-axis position determined by the feature, the x-axis position by the SHAP value, and the color by the size of the feature value. A higher SHAP value indicates that the corresponding sample is more likely to be identified by the model as having a positive outcome. The numerical values in the figure represent the mean absolute SHAP values for each feature. **(A)** ROC curve of the optimal predictive model of baseline disease activity scores; **(B)** ROC curve of the optimal predictive model of treatment response improvement; **(C)** SHAP summary plot of the optimal predictive model of baseline disease activity scores; **(D)** SHAP summary plot of the optimal predictive model of treatment response improvement.

To address multicollinearity among correlated disease activity scores, we employed interpretable machine learning for feature selection. Multiple XGBoost models predicting dupilumab discontinuation were developed using two distinct feature sets: baseline disease activity scores (EASI, SCORAD, DLQI/CDLQI, POEM, NRS, SLS, ADCT) and follow-up binary improvement indicators (BSA-75, EASI-75, SCORAD-75, POEM-75, NRS-75, SLS-75, ADCT-75). Optimal models were selected via 10-fold cross-validation, with corresponding ROC curves shown in **Figures 2A, B**.

SHAP analysis ranked all features by descending mean absolute SHAP values (**Figures 2C, D**), which revealed NRS as the most important PRO feature (mean |SHAP|=0.180) and EASI as the top investigator-assessed feature (mean |SHAP|=0.054). Among treatment responses, EASI-75 (mean |SHAP|=0.049) and SLS-75 (mean |SHAP|=0.047) showed strongest predictive contributions. Pearson correlation analysis (**Table 2**) revealed EASI-75/BSA-75 achievement positively correlated with SHAP values (r>0.8), linking these objective endpoints to higher discontinuation probability. Conversely, achievement of SCORAD-75, ADCT-75, NRS-75 and SLS-75 showed strong negative correlations with SHAP values (r<-0.8). Higher baseline NRS and SLS scores also negatively correlated with SHAP values (r<-0.8), indicating that patients with higher baseline scores for these subjective metrics were less frequently classified as having treatment discontinuation.

**Table 2:** Pearson’s correlation between the values of each feature and the SHAP values.

Feature	r	95% CI
EASI-75	0.950	0.937 to 0.960
BSA-75	0.904	0.881 to 0.923
POEM-75	0.620	0.546 to 0.685
POEM	0.393	0.294 to 0.484
EASI	0.269	0.162 to 0.370
SCORAD	-0.256	-0.357 to -0.148
ADCT	-0.594	-0.662 to -0.517
DLQI	-0.673	-0.730 to -0.607
SLS	-0.857	-0.884 to -0.824
SCORAD-75	-0.877	-0.901 to -0.849
NRS	-0.895	-0.915 to -0.870
ADCT-75	-0.953	-0.962 to -0.941
NRS-75	-0.961	-0.969 to -0.952
SLS-75	-0.973	-0.978 to -0.966

### 3.4 Multivariable Cox proportional hazards model

After feature selection, the multivariable Cox proportional hazards model incorporated key predictors (NRS, EASI, EASI-75, SLS-75) identified through feature engineering and significant univariable analysis variables (age, sex, combination therapy) (**Table 3**). It demonstrated strong discriminative ability (C-index=0.837) without overfitting (Cox-Snell R²=0.396). Based on these, sample size calculation, employing a shrinkage factor of 0.9 and the observed event rate (94/307) as the event rate estimate, indicated minimum requirements of 141 cases for a seven-covariate Cox regression model. In the adjusted model, Younger age, lower baseline NRS and EASI, SLS-75 non-achievement, and EASI-75 achievement independently predicted discontinuation. Sex and combination therapy lost significance after adjusting for disease severity and treatment response.

**Table 3:** Results of the multivariable Cox proportional hazards model.

C-index	SE (C-index)	Cox-Snell R²	Nagelkerke R²	
0.837	0.019	0.396	0.412	
Characteristics	HR	95% CI	P-value	VIF
Sex	1.569	0.891-2.764	0.119	1.250
Age	0.967	0.953-0.982	< 0.001	1.080
Combination therapy	1.023	0.606-1.728	0.931	1.322
EASI	0.980	0.962-0.998	0.028	1.267
NRS	0.820	0.744-0.904	< 0.001	1.307
EASI-75	3.585	1.948-6.598	< 0.001	1.263
SLS-75	0.246	0.120-0.504	< 0.001	1.174

**Figure 3:**
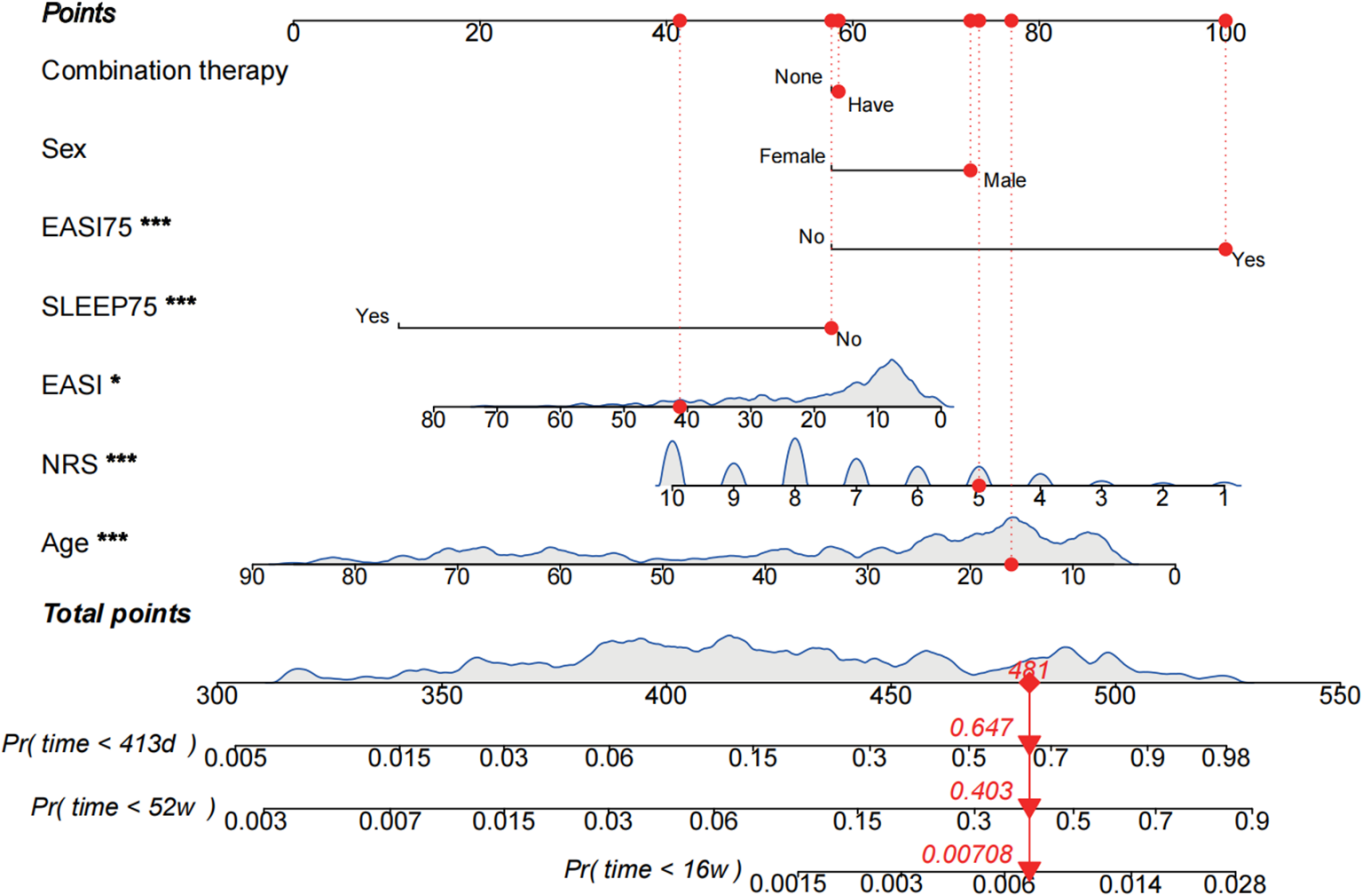
Atopic dermatitis. Nomogram of Cox proportional hazards model for predicting of treatment discontinuation. The nomogram assigns scores to different value levels of each independent variable based on their respective regression coefficients in the model. These variable-specific scores are summed to obtain a total point value. This total is then converted into a predicted probability of the outcome event through a predefined functional mapping relationship. The red marked section shows the characteristic values, corresponding scores and predicted probabilities of the participant #94 (16-year-old male, having combination therapy, EASI = 41.2, NRS = 5, EASI-75 met and SLS-75 unmet) predicted discontinuation probabilities of 0.7%, 40.3%, and 64.7% at 16 weeks, 52 weeks, and 413 days, respectively. *P <0.05, **P <0.01, ***P <0.001.

**Figure 4:**
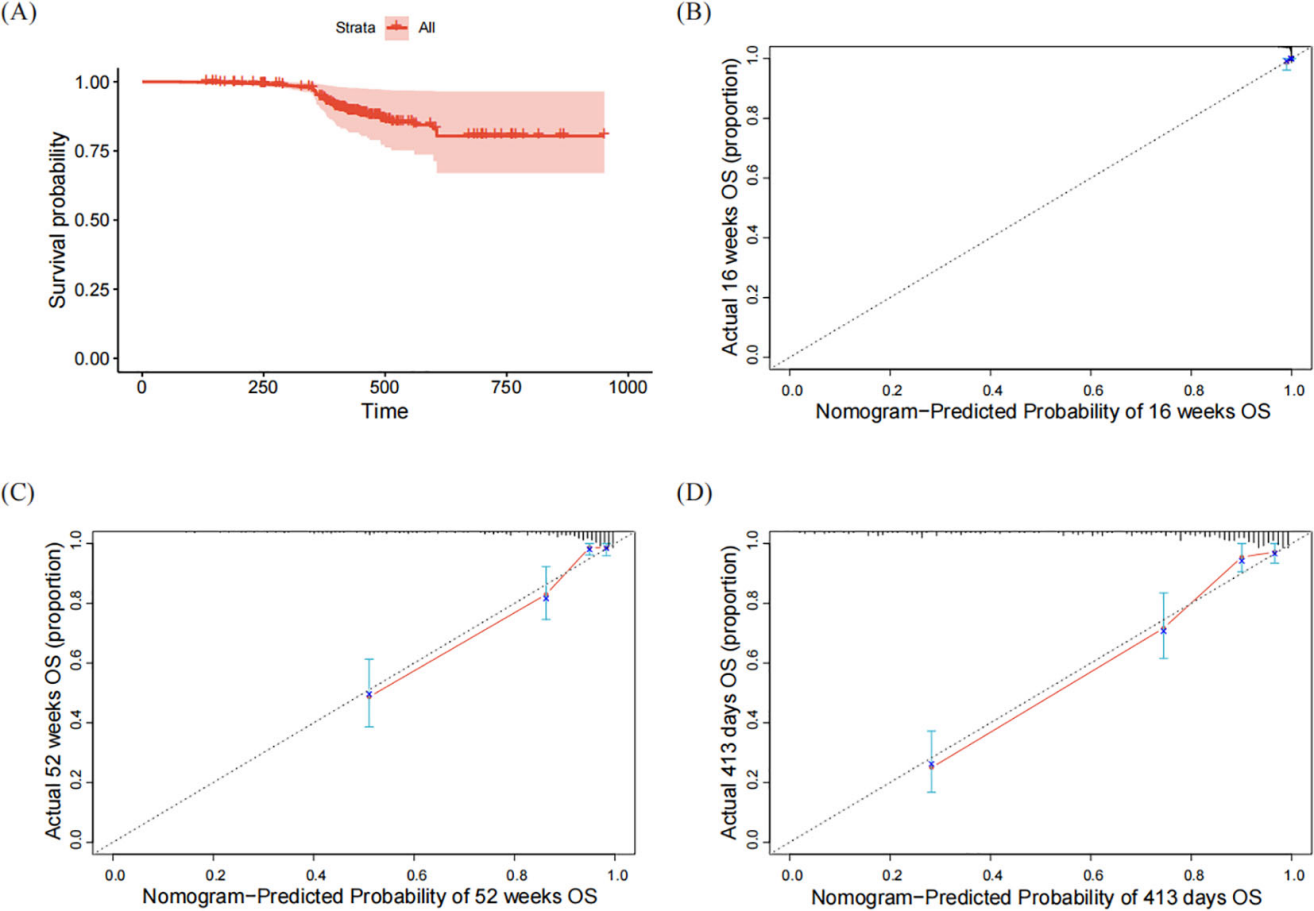
Atopic dermatitis. Kaplan-Meier curve and calibration curves of Nomogram. **(A)** Kaplan-Meier curve; **(B)** Calibration curve for the 16 weeks prediction Nomogram; **(C)** Calibration curve for the 52 weeks prediction Nomogram; **(D)** Calibration curve for the 413 days prediction Nomogram.

Nomograms based on this model could be developed to predict risks at various time points. In this study, nomogram predicted dupilumab persistence at guideline-recommended timepoints (16-week, 52-week) and our cohort’s median duration (413 days), to facilitate identification of new patients at elevated discontinuation risk at our institution (**Figure 3**). For example, in Case #94 (16-year-old male, combination therapy+, EASI = 41.2, NRS = 5, EASI-75+, SLS-75-), predicted discontinuation probabilities were 0.7% (16 weeks), 40.3% (52 weeks), and 64.7% (413 days). This patient subsequently discontinued treatment on day 379 and rejected subsequent treatment. The nomogram shows markedly increased discontinuation risk by week 52, consistent with the survival curve (**Figure 4A**). Calibration curves demonstrated satisfactory prediction-observation agreement (**Figures 4B–D**).

## 4 Discussion

Cox regression associated younger age and earlier age at AD onset with higher dupilumab discontinuation, contrasting with previous reports indicating no association between age and medication adherence (45). In final multivariable Cox regression, age remained significantly associated with adherence after adjusting for other variables, suggesting age-related psychosocial mechanisms beyond clinical factors. Younger patients may show reduced compliance due to limited treatment understanding, while older patients may exhibit enhanced adherence driven by heightened health concerns (21–23). However, age’s effect attenuated after adjustment (HR: 0.958 to 0.967), indicating potential indirect effects through disease severity or treatment response. Current literature lacks cross-age efficacy comparisons, limiting mechanistic exploration (46–48). While the exact mechanisms require further elucidation, closer adherence monitoring for younger patients appears warranted to reduce unnecessary discontinuation and improve long-term control.

Baseline disease activity scores predicted adherence in univariable Cox regression by higher EASI, SCORAD, and PROs scores associated with reduced discontinuation risk. Interpretable machine learning further identified pruritus (NRS) and sleep disruption (SLS) as the two most influential features predicting outcomes. These findings indicate that pre-treatment disease severity substantially influences subsequent adherence patterns, with more severe baseline scores generally predicting better treatment persistence, particularly patient-reported pruritus and sleep disturbance. Meanwhile, PROs demonstrated stronger associations with adherence compared to investigator assessments. The results validate the rationality of the SCORAD scale design, which integrates objective investigator evaluations of lesion characteristics with subjective patient reports of pruritus and sleep disruption (49).

Treatment response analysis revealed a paradoxical pattern: achieving EASI-75/BSA-75 increased discontinuation risk, while achieving SCORAD-75/PROs-75 reduced it. While improved adherence following subjective symptom remission aligns with therapeutic goals, the association between objective sign improvement and elevated discontinuation risk requires explanation. In the absence of established literature, we propose several preliminary hypotheses based on our cohort data. The weaker protective effect of baseline EASI/BSA scores against discontinuation (HR = 0.983-0.995) compared to PROs (HR = 0.691-0.893), with SCORAD intermediate (HR = 0.973), suggests different disease cognition between clinician and patient priorities. This discordance in disease cognition might lead some patients with lower baseline disease severity to consider treatment complete upon achieving EASI-75. Furthermore, the notable discrepancy between EASI-75 achievement (62.2%) and PROs-based improvement rates (25.1%-36.5%) indicates that objective clinical remission often fails to correspond with adequate subjective symptom control. When significant objective improvement coincides with unsatisfactory PROs improvement, patients may perceive diminished treatment value, consequently reducing adherence. These findings collectively indicate a PROs-driven adherence mechanism where treatment decisions prioritize personal symptom experience over objective signs. This paradox underscores a critical gap in current AD assessment, highlighting the urgent need for development of more accurate patient-centered evaluation tools.

For the final multivariable Cox proportional hazards regression model, the C-index and calibration curve performance demonstrated robust discriminative ability and strong clinical applicability. Goodness-of-fit statistics (Cox-Snell R² and Nagelkerke R²) indicated no substantial overfitting. Furthermore, the calculated minimum sample size required for adequate statistical power was considerably lower than the actual sample size utilized for model development. These findings validate the multi-step modeling strategy, which sequentially identified variables with optimal predictive performance, thereby achieving a streamlined yet accurate predictive model. Compared to univariable results, age, NRS, EASI, SLS-75, and EASI-75 remained independently significant, while sex and combination therapy effects were mediated through disease severity and treatment response.

By following the three-stage modeling workflow of this study, the multivariable Cox proportional hazards regression model can be visually presented as a nomogram, enabling prescribing physicians to quantitatively assess the treatment discontinuation risk at critical timepoints in dupilumab therapy: 16-week standard induction phase, 52-week long-term management phase (14–16), and other time points of interest to researchers such as the median treatment duration analyzed in this study. For example, Case #94 (16-year-old male, combination therapy+, EASI = 41.2, NRS = 5, EASI-75+, SLS-75-) showed discontinuation probabilities of 0.7% (16 weeks), 40.3% (52 weeks), and 64.7% (413 days), aligning with actual cessation at day 379. This tool enables dynamic risk estimation using clinical data. When probabilities exceed established thresholds (e.g., ≥50%), enhanced adherence interventions, including developing personalized written treatment plans, providing diversified health education (e.g., face-to-face instruction, brochures, online videos), implementing medication reminders (e.g., phone calls, text messages), and appropriately increasing follow-up frequency (21, 22, 50), can be initiated proactively to improve adherence in AD patients. Over 90% of participants completed the 16-week course, confirming high initial adherence consistent with prior clinical trials (19, 20, 51).

This study has several limitations. First, its single-center design may introduce selection bias. As mentioned in the introduction, treatment adherence is influenced by various factors such as socioeconomic status and cultural practices, which may demonstrate relative homogeneity within our geographically concentrated cohort but differ substantially across other centers. Consequently, the generalizability of our findings requires validation through multi-center studies, which would verify the model’s reliability and broader applicability across diverse clinical settings and populations. Second, the observational design establishes statistical associations but not clear causality, and unmeasured confounders, such as patient heterogeneity, physician communication, may interfere in observational framework. Furthermore, Our hypotheses remain preliminary due to limited existing literature, requiring validation through randomized controlled trials. Additionally, as the cohort primarily included patients receiving long-term therapy due to more complete follow-up data, the findings are most applicable to long-term dupilumab therapy.

## 5 Conclusion

In summary, this single-center study of 307 AD patients identified younger age, lower baseline disease activity, and dissociation between EASI-75 achievement and PROs-75 non-achievement as key predictors of dupilumab discontinuation. These findings highlight the dominant role of patient-reported symptoms in adherence behavior and the need to balance objective signs with subjective experiences like pruritus and sleep disturbance. The machine learning-enhanced Cox model maintained predictive accuracy with reduced variables, while the resulting nomogram offers clinicians a practical tool for personalized risk assessment and intervention.

## Data Availability

The raw data supporting the conclusions of this article will be made available by the authors, without undue reservation.
