# Supplementary material for: Influencing factor analysis and prediction model construction of dupilumab treatment adherence: a prospective cohort study in moderate-to-severe atopic dermatitis
Source: Front Immunol. 2026 Jan 5;16:1682777. doi: 10.3389/fimmu.2025.1682777 (PMC12813149; doi:10.3389/fimmu.2025.1682777)
Supplement: Supplementary file 1 [file Table1.docx]

**Supplementary Table 1. Detailed description of comorbidities, combination therapy and treatment interval.**

| **Characteristics** | **Participants (N=307)** |
| --- | --- |
| Comorbidity, n (%) | 149 [48.5] |
| Comorbidity group, n (%) |  |
| Allergic rhinitis | 105 [34.2] |
| Asthma | 12 [3.9] |
| Urticaria | 7 [2.3] |
| Conjunctivitis | 9 [2.9] |
| Above-mentioned two kinds | 15 [4.9] |
| Above-mentioned more kinds | 1 [0.3] |
| Combination therapy, n (%) | 119 [38.8] |
| Combination therapy group, n (%) |  |
| Glucocorticoids | 44 [14.3] |
| PDE4 inhibitors | 14 [4.6] |
| Calcineurin inhibitors | 4 [1.3] |
| Antihistamines | 13 [4.2] |
| JAK inhibitors | 4 [1.3] |
| Traditional Chinese medicine | 5 [1.6] |
| Above-mentioned two kinds | 23 [7.5] |
| Above-mentioned more kinds | 12 [3.9] |
| Treatment interval (weeks), median [Q1-Q3] | 2 [2-4] |
| Treatment interval (weeks) group, n (%) |  |
| 2 | 199 [64.8] |
| 3 | 29 [9.4] |
| 4 | 67 [21.8] |
| 5 to < 8 | 6 [2.0] |
| ≥ 8 | 6 [2.0] |
