# Supplementary material for: Influencing factor analysis and prediction model construction of dupilumab treatment adherence: a prospective cohort study in moderate-to-severe atopic dermatitis
Source: Front Immunol. 2026 Jan 5;16:1682777. doi: 10.3389/fimmu.2025.1682777 (PMC12813149; doi:10.3389/fimmu.2025.1682777)
Supplement: Supplementary file 2 [file Table2.docx]

**Supplementary Table 2. Results of the univariable Cox proportional hazards model between single variables and treatment discontinuation.**

| **Characteristics** | **HR** | **95% CI** | **P-value** |
| --- | --- | --- | --- |
| Sex | 2.770 | 1.673-4.587 | < 0.001 |
| Age | 0.958 | 0.945-0.972 | < 0.001 |
| Age at AD onset | 0.968 | 0.956-0.981 | < 0.001 |
| Family history | 1.146 | 0.758-1.735 | 0.518 |
| Comorbidity | 1.358 | 0.902-2.045 | 0.143 |
| Combination therapy | 0.527 | 0.335-0.829 | 0.006 |
| Treatment interval | 1.035 | 0.960-1.116 | 0.373 |
| IGA | 0.853 | 0.585-1.242 | 0.406 |
| BSA | 0.995 | 0.987-1.004 | 0.266 |
| EASI | 0.983 | 0.968-0.999 | 0.034 |
| SCORAD | 0.973 | 0.961-0.985 | < 0.001 |
| DLQI/CDLQI | 0.873 | 0.839-0.909 | < 0.001 |
| POEM | 0.893 | 0.861-0.927 | < 0.001 |
| NRS | 0.691 | 0.635-0.751 | < 0.001 |
| SLS | 0.739 | 0.686-0.797 | < 0.001 |
| ADCT | 0.863 | 0.833-0.895 | < 0.001 |
| IGA 0/1 | 0.676 | 0.326-1.404 | 0.294 |
| BSA-75 | 1.622 | 1.053-2.498 | 0.028 |
| EASI-75 | 3.335 | 1.946-5.715 | < 0.001 |
| SCORAD-75 | 0.180 | 0.066-0.491 | 0.001 |
| DLQI-75/CDLQI-75 | 0.750 | 0.461-1.219 | 0.246 |
| POEM-75 | 0.353 | 0.200-0.624 | < 0.001 |
| NRS-75 | 0.111 | 0.045-0.275 | < 0.001 |
| SLS-75 | 0.180 | 0.093-0.347 | < 0.001 |
| ADCT-75 | 0.161 | 0.078-0.332 | < 0.001 |
